# Oscillatory Reinstatement Enhances Declarative Memory

**DOI:** 10.1523/JNEUROSCI.0265-17.2017

**Published:** 2017-10-11

**Authors:** Amir-Homayoun Javadi, James C. Glen, Sara Halkiopoulos, Mei Schulz, Hugo J. Spiers

**Affiliations:** ^1^School of Psychology, Keynes College, University of Kent, Canterbury CT2 7NP, United Kingdom, and; ^2^Institute of Behavioural Neuroscience, Department of Experimental Psychology, University College London, London WC1H 0AP, United Kingdom

**Keywords:** context, dorsolateral prefrontal cortex (DLPFC), memory, oscillation, reinstatement, transcranial alternating current stimulation (tACS)

## Abstract

Declarative memory recall is thought to involve the reinstatement of neural activity patterns that occurred previously during encoding. Consistent with this view, greater similarity between patterns of activity recorded during encoding and retrieval has been found to predict better memory performance in a number of studies. Recent models have argued that neural oscillations may be crucial to reinstatement for successful memory retrieval. However, to date, no causal evidence has been provided to support this theory, nor has the impact of oscillatory electrical brain stimulation during encoding and retrieval been assessed. To explore this we used transcranial alternating current stimulation over the left dorsolateral prefrontal cortex of human participants [*n* = 70, 45 females; age mean (SD) = 22.12 (2.16)] during a declarative memory task. Participants received either the same frequency during encoding and retrieval (60–60 or 90–90 Hz) or different frequencies (60–90 or 90–60 Hz). When frequencies matched there was a significant memory improvement (at both 60 and 90 Hz) relative to sham stimulation. No improvement occurred when frequencies mismatched. Our results provide support for the role of oscillatory reinstatement in memory retrieval.

**SIGNIFICANCE STATEMENT** Recent neurobiological models of memory have argued that large-scale neural oscillations are reinstated to support successful memory retrieval. Here we used transcranial alternating current stimulation (tACS) to test these models. tACS has recently been shown to induce neural oscillations at the frequency stimulated. We stimulated over the left dorsolateral prefrontal cortex during a declarative memory task involving learning a set of words. We found that tACS applied at the same frequency during encoding and retrieval enhances memory. We also find no difference between the two applied frequencies. Thus our results are consistent with the proposal that reinstatement of neural oscillations during retrieval supports successful memory retrieval.

## Introduction

Declarative memory recall is thought to involve the reinstatement of neural activity patterns that occurred previously during encoding (Norman and O'Reilly, 2003; Teyler and Rudy, 2007). In agreement with this model, greater similarity between patterns of activity recorded during encoding and retrieval tends to predict better memory performance (Polyn et al., 2005; Johnson et al., 2009; Gordon et al., 2014). Recent models, drawing on new findings (Sederberg et al., 2007a; Wimber et al., 2012), have argued that neural oscillations may be crucial to reinstatement (Watrous and Ekstrom, 2014). However, to date, no causal evidence has been provided to support this theory, nor has the impact of oscillatory electrical brain stimulation during encoding and retrieval been assessed.

We now report causal evidence in support of the oscillatory reinstatement hypothesis. We took advantage of the potential capacity of transcranial alternating current stimulation (tACS) to entrain oscillations (Zaehle et al., 2010; Helfrich et al., 2014a). Gamma and theta oscillations have been shown to have a mechanistic role in memory formation (Sederberg et al., 2003, 2007b), linking memory formation to cellular mechanisms of learning (Jutras and Buffalo, 2010), and coordination of hippocampus with other brain areas (Fell et al., 2001; Colgin, 2011; for review see, Igarashi et al., 2014). We focused on gamma oscillations because tACS has been shown to enhance gamma oscillations (Strüber et al., 2014; Helfrich et al., 2014b), whereas tACS at other lower frequencies has been shown to have mixed effects on oscillatory power (Veniero et al., 2015). Additionally, as compared with theta band which has a narrower frequency band (4–7 Hz), gamma band have a wide frequency range of 30–120 Hz, which gave us the flexibility of selecting two frequencies that are separate enough (i.e., 60 and 90 Hz). We applied tACS to the left dorsolateral prefrontal cortex (DLPFC) of participants during a declarative memory task. The left DLPFC was targeted due to past research showing successful modulation of declarative memory by electrical brain stimulation (Javadi and Walsh, 2012; Javadi et al., 2012; Manenti et al., 2013; Sandrini et al., 2014; Zwissler et al., 2014). Participants received either the same frequency during encoding and retrieval (congruent group) or different frequencies (incongruent group). In a separate session, one week apart, sham stimulation was applied during encoding and retrieval. The order of sham and stimulation sessions was counter balanced across participants. Memory accuracy in this session was used as the baseline to compare with memory accuracy in the active-stimulation session.

## Materials and Methods

### 

#### 

##### Participants.

**Figure 1.**
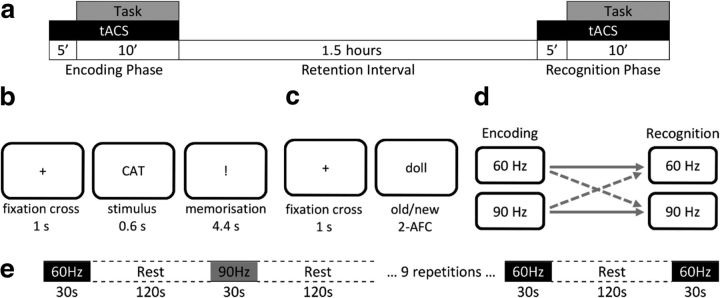
***a***, Procedure of an experimental day. ***b***, Procedure of the encoding phase. ***c***, Procedure of the recognition phase. ***d***, Solid and dashed lines represent congruent and incongruent conditions, respectively. Participants took part in two sessions with either active or sham stimulation conditions. ***e***, Protocol of the control study, showing a sample sequence of the two stimulation protocols. With the exception of the first episode of stimulation, after each stimulation episode participants were asked to judge whether the last episode was the “same” or “different” to the previous one. The combination of stimulation and rest was repeated nine times in total, yielding eight comparisons. The order was pseudorandomly arranged such that there were four comparisons where stimulation was at the same frequency and four that were different.

Seventy healthy native English speaking adults [45 females, age mean (SD) = 22.12 (2.16)] took part in two experimental sessions. They were randomly assigned to one of four conditions based on the frequency of tACS administered during encoding and retrieval. Congruent groups were 60–60 (*n* = 17) and 90–90 (*n* = 18), and incongruent groups were 60–90 (*n* = 17) and 90–60 (*n* = 18). For example, 60–90 indicates an incongruent condition with 60 Hz tACS during encoding and 90 Hz tACS during retrieval (Fig. 1*d*).

All participants were naive to the study, English first language speakers, and right-handed yielding a laterality quotient of at least +50 on the Edinburgh Handedness Inventor (Oldfield, 1971). All participants had normal or corrected-to-normal vision, and all were screened to exclude those with a history of neurologic trauma or psychiatric disorder. No participant was taking any centrally acting medications. All participants gave their written informed consent in accordance with the Declaration of Helsinki and the guidelines approved by the Ethical Committee of University College London.

##### Transcranial alternative current stimulation.

tACS (neuroConn DC Brain Stimulator Plus, neruoCare) was administered via two 5 × 7 cm^2^ saline-soaked surface sponge electrodes. One electrode was placed over the left DLPFC (F3 according to the 10–20 international system for EEG electrode placement) and one electrode over the left wrist. The left DLPFC was stimulated because both functional neuroimaging and brain stimulation data indicate it plays a prominent role in memory processing of written words (Blumenfeld and Ranganath, 2006; Staresina and Davachi, 2006; Murray and Ranganath, 2007; Javadi and Walsh, 2012; Javadi et al., 2012; Javadi and Cheng, 2013; for reviews of prefrontal interactions in long-term memory, see Simons and Spiers, 2003; Blumenfeld and Ranganath, 2007).

tACS was delivered during both encoding and retrieval phases with 1.5 mA peak-to-peak amplitude and 1 s ramp up and down. Stimulation was delivered either at 60 or 90 Hz. These specific gamma frequencies were chosen because they both fall in the high gamma range (60–140 Hz), they are not resonant harmonics of one another, and there is a reasonable separation between them. This ensured that when stimulating at one of these frequencies the effect on the other frequency was minimized.

In both encoding and retrieval phases of one session, participants were stimulated for 15 min or 16 s for active (60–60, 90–90, 60–90, and 90–60) and sham stimulation conditions, respectively.

##### Stimuli.

A bank of 590 words was extracted from The MRC psycholinguistic database (Coltheart, 1981). Words with high valence or similar meanings were excluded. Given that participants were instructed to visualize the words, highly familiar and easily imaginable words were selected. The words were controlled for the number of letters [minimum = 3, maximum = 8, mean (SD) = 4.89 (1.24)], number of syllables [minimum = 1, maximum = 2, mean (SD) = 1.49 (0.50)], printed familiarity [mean (SD) = 552.54 (34.75)], concreteness [mean (SD) 580.60 (34.06)], and imaginability [mean (SD) = 581.73 (33.20)]. For each participant, the words used differed on each day.

##### Procedure.

Each participant took part in 2 experimental days: one active stimulation and one sham stimulation, 1 week apart. Active and sham stimulation days were counterbalanced across participants. Participants were told that they both days would follow the same procedure and task. Each day consisted of encoding and retrieval phases and a 1.5 h retention interval in which participants stayed in the laboratory. During this time they watched episodes of a TV series while refraining from alcohol, caffeine, and smoking (Fig. 1*a*). Participants were aware that they would be tested on their memory performance in the retrieval session.

Behavioral tasks began 5 min after the onset of stimulation. In the encoding phase 100 words were presented one at a time (0.6 s) followed by an exclamation mark presented for 4.4 s which was designated as the memorization period. Words were separated by a 1 s fixation cross (Fig. 1*b*). During the retrieval phase, previously presented words were randomly interleaved with 100 new words and presented one at a time. Participants were asked to judge whether the presented word was old (studied during the encoding phase) or new (Fig. 1*c*). The retrieval session was self-paced, but participants were told to work as swiftly as they could without sacrificing accuracy.

Stimulus presentation and response recording was conducted using MATLAB v2013b (MathWorks) and the Psychophysics Toolbox v3 (Brainard, 1997). SPSS v21 (IBM Lead Technologies) was used to carry out statistical analysis on the data.

##### Control study.

To investigate whether participants in the incongruent condition could discriminate between the two stimulation frequencies, we ran a control study. Eighteen participants [10 female, mean age (SD) = 23.42 (3.18)] took part in this study. During a randomized sequence of stimulation episodes (either 60 or 90 Hz), participants were asked to judge whether the stimulation was the “same” or “different” from the previous episode. Stimulation setup was similar to the main study, except it was delivered for only 30 s, followed by a 2 min rest. This was repeated nine times resulting in eight responses (no judgment was made after the first session as there was no comparison). See Figure 1*e* for an example stimulation sequence.

##### Statistical data analysis.

Response accuracy and reaction time were recorded for data analysis. Trials with response times <200 and >;5000 ms were removed from analysis (0.128% of whole data).

We analyzed the data based on accurate responses for old and new words, as well as *d′. d′* itself was calculated as *z*(Hits) − *z*(False Alarms). To determine whether left DLPFC tACS modulated memory performance we calculated the difference in performance between active and sham stimulation conditions (“percentage accuracy difference” for percentage accuracy and “*d′* difference” for *d′*). We used performance difference because it provides measure of how much each individual was affected by the stimulation, thus accounting for variation between subjects in their sham session performance. Three 2 × 2 ANOVAs were conducted with frequency at the first and second sessions (60 and 90 Hz) as independent factors, and difference in percentage accuracy for old and new words, and *d′* difference as dependent factors in each ANOVA. *Post hoc* one-sample *t* tests were run to compare percentage accuracy difference and *d′* difference with zero in different groups. Effect sizes partial-η squared for ANOVA and Cohen's *d* are reported. Similar analyses were conducted on response times.

Data from the control study was analyzed using a one-sample *t* test comparing the mean performance accuracy with chance level 50%.

## Results

**Figure 2.**
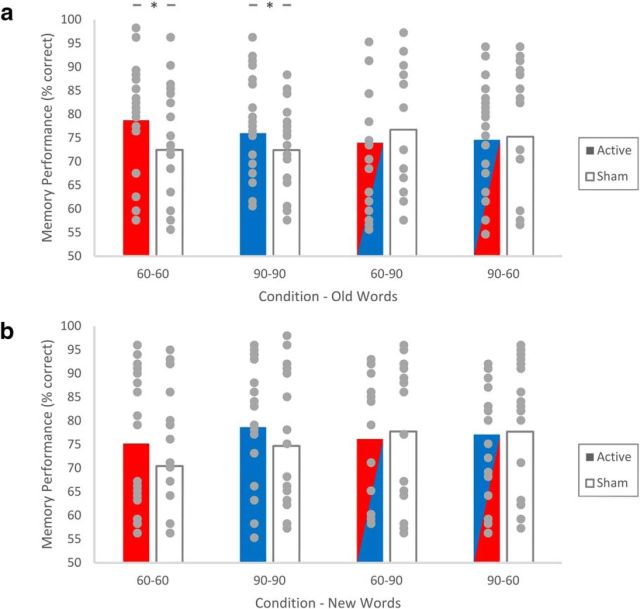
Performance (percentage correct for both old and new words) of the participants (within-subject design) in active and sham stimulation conditions for (***a***) old and (***b***) new words. Paired-sample *t* tests comparing performance between active and sham stimulation conditions. Circles represent individual data points. *p < 0.05.

In this study tACS was applied over the scalp above the left DLPFC of participants in a frequency-specific manner during a task involving encoding and delayed retrieval of written words. The ANOVAs looking at percentage accuracy difference for both old and new words, as well as *d′* difference, revealed nonsignificant main effects of frequency in the first and second sessions, but a significant interaction effect (Table 1). *Post hoc* one-sample *t* tests on percentage accuracy difference showed significant differences for both of the congruent conditions for old words, and not for the conditions relating to new words (Fig. 2; Table 2). Similar tests on *d′* difference revealed that both congruent stimulation groups showed a significant enhancement in performance relative to sham stimulation, but no significant effect was present for either of the two incongruent groups (Table 3).

**Table 1.** Summary of the 2 × 2 ANOVA with frequency of the 1st and 2nd sessions (60 and 90 Hz) as independent factors, and percentage accuracy difference (percentage accuracy for the active − sham stimulation condition) for the old words and new words (separate), and *d′* difference (*d′* for the active − sham stimulation condition) as dependent factors

Effect	Accuracy difference for old words, %			Accuracy difference for new words, %			*d′* difference		
	*F*	*p*	η_*p*_^2^	*F*	*p*	η_*p*_^2^	*F*	*p*	η_*p*_^2^
Main effect of 1st session	0.245	0.622	0.004	0.002	0.963	<0.001	1.631	0.206	0.024
Main effect of 2nd session	2.706	0.105	0.039	0.138	0.711	0.002	2.769	0.101	0.040
Interaction	11.681	0.001*	0.150	5.383	0.023*	0.75	9.974	0.002*	0.131

*F*_(1, 70)_ for all effects.

*p < 0.05.

**Table 2.** Summary of *post hoc* one-sample *t* test on percentage accuracy difference (percentage accuracy for the active − sham stimulation condition) for the old and new words separately

Condition, Hz	Accuracy difference for old words, %			Accuracy difference for new words, %		
	*t*	*p*	*d*	*t*	*p*	*d*
60–60; *t*_(16)_	2.815	0.012*	0.269	1.785	0.093	0.159
90–90; *t*_(17)_	2.261	0.037*	0.210	1.515	0.148	0.011
60–90; *t*_(16)_	−1.737	0.102	0.173	−0.858	0.404	0.048
90–60; *t*_(17)_	−0.411	0.686	0.108	−0.284	0.779	0.191

*d*, Cohen's *d* effect size.

*p < 0.05.

**Table 3.** Summary of *post hoc* one-sample *t* test on *d*′

Condition, Hz	Active	Sham	*t*	*p*	*d*
60–60; *t*_(16)_	1.71 (1.04)	1.31 (0.94)	2.583	0.020*	0.403
90–90; *t*_(17)_	1.85 (0.89)	1.50 (0.83)	2.705	0.015*	0.406
60–90; *t*_(16)_	1.50 (0.99)	1.78 (1.11)	−1.751	0.099	0.479
90–60; *t*_(17)_	1.80 (0.96)	1.66 (0.97)	0.957	0.352	0.043

The raw values of *d′* are shown as mean (SD).

*d*, Cohen's *d* effect size.

*p < 0.05.

Although tACS above the left DLPFC appears to affect memory accuracy, no such effects were found for reaction times (RTs). Examining the differences between mean RTs in active and sham conditions for old and new words using two 2 × 2 ANOVAs revealed no significant main effects, nor significant interactions (all *p* values >;0.200).

Analysis of the control study showed that performance was not above chance level (*t*_(17)_ = 1.22, *p* = 0.23). This nonsignificant difference shows that participants were not able to discriminate between the two stimulation protocols.

## Discussion

In summary, relative to sham stimulation, memory performance (both when measured by percentage accuracy for old words and *d′*) was significantly enhanced only when the same frequency was applied at encoding and retrieval (at both 60 and 90 Hz), no significant effects were observed when the frequency of stimulation differed between encoding and retrieval. This effect was specific to performance, with no improvement in reaction times observed. Our analysis revealed that these effects were specifically present when individual subject's performance is accounted for by using our within-subjects measure of performance difference.

Using the capacity of tACS to entrain oscillations in the cortex (Zaehle et al., 2010; Helfrich, Schneider et al., 2014) we test three hypotheses: (1) application of oscillatory brain stimulation during encoding and retrieval enhances memory, (2) memory performance may be enhanced by specific stimulation frequencies, and (3) the reinstatement of the same frequency is required across encoding and retrieval for memory enhancement. Our first hypothesis was motivated by the possibility that tACS provides a similar enhancing effect on memory as transcranial direct current stimulation (tDCS). This is based on evidence that both tDCS and tACS can enhance working memory (Fregni et al., 2005; Andrews et al., 2011; Jaušovec and Jaušovec, 2014) and that tDCS can enhance declarative memory (Jacobson et al., 2012; Javadi and Walsh, 2012; Javadi et al., 2012; Javadi and Cheng, 2013). Thus, it seems plausible that tACS, like tDCS, may also generally enhance declarative memory. Motivation for our second hypothesis comes from evidence that power increases in certain gamma frequency bands (30+ Hz) have consistently been associated with successful memory retrieval (Gruber et al., 2004; Osipova et al., 2006; Sederberg et al., 2007a; Hanslmayr and Staudigl, 2014; for review, see Düzel et al., 2010; Nyhus and Curran, 2010), suggesting that certain frequencies of tACS may be more beneficial than others. Our third hypothesis is based on recent models (Hanslmayr and Staudigl, 2014; Watrous and Ekstrom, 2014) that argue that frequency-specific reinstatement of oscillatory activity from encoding during retrieval serves accurate memory recall.

Our results help advance models of memory processing. Numerous models have argued that the successful retrieval of past experience involves a reinstatement of activity that previously occurred during encoding (Norman and O'Reilly, 2003; Teyler and Rudy, 2007). More recently several models have argued that neural oscillations are central to this reinstatement process (Watrous and Ekstrom, 2014; Watrous et al., 2015). Such models build on mounting evidence that memory retrieval success is related to the strength of the correlation between neural oscillations recorded during encoding and retrieval (Sederberget al., 2007a; Wimber et al., 2012). However, it has been noted that causal evidence for this process is lacking (Sederberg et al., 2007a; Watrous et al., 2015). Thus, our evidence that oscillatory brain stimulation (tACS) at the same frequency at encoding and retrieval leads to an enhancement of memory provides important support for these theories. Neural recording studies showing oscillatory reinstatement demonstrate that the reinstatement can occur very rapidly after cueing (Wimber et al., 2012), leading to the suggestion that the reinstatement is part of the rapid retrieval process, rather than post-retrieval processing. In this view, the stimulation enhances memory by reinstating the encoding conditions in the network of brain areas responsible for the reactivation of the memory trace. This reinstatement may enhance processes such as pattern completion (Staresina et al., 2012, 2016; Tompary et al., 2016) where similar network level inputs are transmitted to regions reconstructing the pattern of activity laid down at encoding, such as is thought to occur in hippocampal area CA3 (Norman and O'Reilly, 2003; Neunuebel and Knierim, 2014; Rolls, 2016). However, it is also possible that oscillatory reinstatement aids memory performance by enhancing a post-retrieval process where the oscillations help mediate interactions between different brain areas (Watrous et al., 2013; Thakral et al., 2015). For example, a potential mechanism might be that after the memory trace is reactivated the reinstated oscillations from the time of encoding provide extra neural context (specific network patterns of activity) that improves the assessment of retrieved memory to determine veracity of the memory. Future work will be required to clarify such aspect of the models.

Our results showed that superior performance in the congruent stimulation condition as compared with incongruent stimulation condition was due to better recognition of old stimuli (Table 2). This suggests the enhancement may be more specific to retrieving the memory trace rather than enhancing post-retrieval processes that allow new items to be rejected (Javadi and Walsh, 2012; Manenti et al., 2013; Chua and Ahmed, 2016). Given evidence of two distinct but interacting functional systems for familiarity and novelty (Tulving et al., 1996; Viskontas et al., 2006; Yassa and Stark, 2008; Kafkas and Montaldi, 2014) another possibility is that the specific enhancement for “old” judgements is due to an upregulation in the familiarity system. It may be that targeting the DLPFC with our stimulation had this effect due to the involvement of prefrontal regions in supporting familiarity judgments (Tulving et al., 1996; Yassa and Stark, 2008; Kafkas and Montaldi, 2014). Further research separating novel, familiarity, and recollection would be useful to explore these possibilities.

Our results provide a novel addition to research into state-dependent learning, which has predominantly been investigated via the application of drugs during encoding and retrieval (Goodwin et al., 1969; Petersen, 1979; Kelemen and Creeley, 2003). Such prior work has shown that matching physiological states during encoding and retrieval can enhance memory, but there has been little attempt to manipulate the specific neural mechanisms underlying state-dependent memory. By building on recent observations that tACS can entrain brain oscillations (Helfrich et al., 2014a), we were able to specifically test model predictions (Watrous and Ekstrom, 2014; Watrous et al., 2015) and the specificity of the entrainment. One possible outcome from our results would have been that the state-dependent induction of gamma oscillations is sufficient to enhance memory. However we did not find this, rather we found that a precise match in the exact gamma frequency used was required to induce our memory enhancement, as predicted by models (Watrous and Ekstrom, 2014). Our results cannot be explained by the subjective discrimination between the two stimulation frequencies, as our control study showed that participants were not able to judge whether two consecutively delivered stimulations are the same or different. This supports the contention that impact of tACS is due to alterations in brain oscillations rather than an awareness of being in different states.

Previous studies have shown memory enhancement after deep brain stimulation to the entorhinal cortex (Cheng and Anderson, 2012; Suthana et al., 2012), transcranial magnetic stimulation to scalp above the DLPFC (Turriziani et al., 2012) or parietal cortex (Wang et al., 2014), and tDCS to the scalp above DLPFC (Javadi and Walsh, 2012; Javadi and Cheng, 2013; for reviews, see Spiers and Bendor, 2014; Spiers et al., 2017). Here we extend such work to the relatively less studied application of tACS, and in contrast to the majority of electrical stimulation studies (Bestmann et al., 2015), we provide a test of a specific neural mechanism—oscillatory reinstatement. Although our stimulation protocol targeted the left DLPFC, it is important to note that the relatively wide spatial extent of tACS means that it may have had effected adjacent brain regions. In addition, DLPFC has many connections with both cortical and subcortical structures; therefore, although stimulation occurred here there may have been effects at remote sites. As such, caution must be taken when interpreting the specificity of our observed results to the left DLPFC. One useful extension of our findings is to the domain of memory consolidation, where a number of studies have begun to explore the impact of oscillatory brain stimulation during sleep (Marshall et al., 2006, 2011). In this context it is possible that reinstatement of the gamma frequencies used in our study may disrupt the endogenous slow-wave sleep period consolidation mechanisms leading to memory deficits and perhaps the introduction of previous waking experiences into the post-sleep dream reports through the reactivation of memory traces.
